# Are do-not-resuscitate orders associated with limitations of care beyond their intended purpose in patients with acute intracerebral haemorrhage? Analysis of the ABC-ICH study

**DOI:** 10.1136/bmjoq-2020-001113

**Published:** 2021-02-04

**Authors:** Jatinder S Minhas, Camilla Sammut-Powell, Emily Birleson, Hiren C Patel, Adrian R Parry-Jones

**Affiliations:** 1 Cardiovascular Sciences, University of Leicester, Leicester, UK; 2 Centre for Health Informatics, The University of Manchester, Manchester, Manchester, UK; 3 Manchester Centre for Clinical Neurosciences, Salford Royal NHS Foundation Trust, Salford, Salford, UK; 4 Division of Cardiovascular Sciences, The University of Manchester, Manchester, UK

**Keywords:** resuscitation, palliative care, cardiopulmonary resuscitation, healthcare quality improvement, hospital mortality

## Abstract

Implementation of an acute bundle of care for intracerebral haemorrhage (ICH) was associated with a marked improvement in survival at our centre, mediated by a reduction in early (<24 hours) do-not-resuscitate (DNR) orders. The aim of this study was to identify possible mechanisms for this mediation. We retrospectively extracted additional data on resuscitation attempts and supportive care. This observational study utilised existing data collected for the Acute Bundle of Care for ICH (ABC-ICH) quality improvement project between from 2013 to 2017. The primary outcome was whether a patient received an early (<24 hours) DNR order. We used multivariable logistic regression to estimate the adjusted association between clinically meaningful factors, including an indicator for a change in treatment on the introduction of the ABC care bundle. Early DNR orders were associated with a reduced odds of escalation to critical care (OR: 0.07, 95% CI: 0.03 to 0.17, p<0.001). Commencement of palliative care within 72 hours was far more likely (OR: 8.76, 95% CI: 4.74 to 16.61, p<0.001) if an early DNR was in place. The cardiac arrest team were not called for an ICH patient before implementation but were called on five occasions overall during and after implementation. Further qualitative evaluation revealed that on only one occasion was there a cardiac or respiratory arrest with cardiopulmonary resuscitation performed. We found no significant increase in resuscitation attempts after bundle implementation but early DNR orders were associated with less admission to critical care and more early palliation. Early DNR orders are associated with less aggressive supportive care and should be judiciously used in acute ICH.

## Introduction

Intracerebral haemorrhage (ICH) has a 1-month case-fatality of 30% to 40% and only one-fifth regain independence.^1^ Globally, haemorrhagic stroke accounts for almost half of 6.5 million annual stroke deaths.^2^ Few effective treatments exist and clinicians may be overly pessimistic when managing ICH patients,^3^ but rapid reversal of anticoagulation,^4–6^ intensive blood pressure (BP) lowering^7^ and neurosurgery can improve outcomes.^8^ The American Heart Association/American Stroke Association 2015 Guidelines for the Management of Spontaneous ICH encourage aggressive early care and postponement of new do-not-resuscitate (DNR) orders until the second full day of hospital admission.^9^

A quality improvement programme, Acute Bundle of Care for ICH (ABC-ICH), was conducted at Salford Royal Hospital, a large Comprehensive Stroke Centre in the UK. To facilitate implementation, ABC-ICH combined recommended care as the ‘ABC bundle’ and provided evidence that the delivery of this bundle improved survival with a 44% relative reduction in 30-day case fatality after implementation, versus before.^10^ The effect of the care bundle was partially mediated by a reduction in early (<24 hours) DNR orders (52.8%) and increased admission to critical care (11.1%).^10^ The mechanisms underpinning the mediating effect of early DNR orders is uncertain. Through secondary analyses of the ABC-ICH dataset, we sought to further understand this observation.

Our objectives were:

1. Determine which baseline and disease severity factors were associated with the use of early DNR orders.
2. Evaluate the frequency and outcome of resuscitation attempts in patients without DNR orders before, during and after the quality improvement project.
3. Determine whether early DNR orders after admission to hospital were associated with limitations in care, after adjusting for baseline factors and ICH severity.

## Methods

This retrospective observational study used existing data collected for the ABC-ICH quality improvement project between 1 June 2013 and 31 May 2017 at Salford Royal Hospital, as described in detail elsewhere.^10^ The ABC care bundle comprises evidence-based interventions recommended in guidelines^9 11^ including process targets for anticoagulant reversal, intensive BP lowering and adherence to a care pathway for neurosurgical referral. Consecutive, spontaneous ICH cases were collected during the study period. Patients with traumatic ICH, haemorrhagic transformation of ischaemic stroke and primary subdural or subarachnoid haemorrhage were excluded. The study was characterised by three periods: before (1 June 2013 to 31 May 2015), during (1 June 2015 to 31 May 2016) and after (1 June 2016 to 31 May 2017) implementation of the ABC care bundle. The DNR policy applies only to the cardiopulmonary resuscitation (CPR) procedure that is followed if there is cessation of breathing (respiratory arrest) and/or a patient’s heart stops beating (cardiac arrest) and should not be linked to any other medical or nursing care, such as the administration of fluids, antibiotics or medications for symptom control. Baseline characteristics, clinical presentation, acute care processes, clinical observations and diagnostic brain imaging characteristics were extracted from the electronic patient record (EPR). Brain scans were reviewed by experienced stroke clinicians to determine ICH location, intraventricular haemorrhage and haematoma volume using the well validated length, width and height (ABC/2) method.^12^ Early neurological decline (END) was defined as a drop in Glasgow Coma Scale (GCS) ≥2 and/or an increase in the National Institutes of Health Stroke Scale score ≥4 occurring within 48 hours of onset, lasting 8 hours, leading to surgery or leading to death.

For this secondary analysis, we identified any cardiac arrest calls occurring in ICH patients during the study period and extracted qualitative data on the event leading to the call, its management and outcome. We used multivariable logistic regression to estimate the adjusted association between early DNR orders and admission before, during or after the ABC-ICH project, adjusting for demographic, premorbid, clinical and radiological factors. We then tested for associations between early DNR orders and the following care processes: anticoagulant reversal (door-to-needle), BP lowering (door-to-target), admission to critical care <72 hours, repeat scan <6 hours of END and commencement of end of life care <72 hours. For patients missing their door-to-needle or door-to-target times, a value of 6 hours was used. For continuous outcomes, multivariable linear regression was used to determine the association between an early DNR order and outcome, adjusting for clinically relevant variables. A log-transformation was applied to the outcome, where appropriate. For binary outcomes, multivariable logistic regression was performed.

## Results

The study included 353 patients before, 265 during, and 242 after implementation (table 1). The discrepancy in patient totals is due to an incorrect recording of the date of ICH in a previous version of the data. The majority of patients reported a premorbid modified Rankin Scale (mRS) of 0 and suffered from a deep ICH. The groups differed in spread of premorbid mRS (p=0.027) and time from onset to arrival (p<0.001). However, there were no statistically significant differences in the ICH imaging characteristics (table 1).

**Table 1:** Baseline characteristics, care process measures and outcomes before (1 June 2013 to 31 may 2015), during (1 June 2015 to 31 may 2016) and after (1 June 2016 to 31 may 2017) the introduction of the care bundle at Salford Royal Hospital

	Before	During	After	P value
	N=353	N=265	N=242	
Baseline factors				
Female, n (%)	186 (52.7)	127 (47.9)	128 (52.9)	0.422
Age, mean (SD)	66.9 (16.2)	66.1 (17.1)	69.2 (14.9)	0.078
GCS, median (IQR)	14 (11–15)	14 (11–15)	14 (11–15)	0.472
modified Rankin Scale score, n (%)				**0.027**
0	218 (61.8)	173 (65.3)	183 (75.6)	
1	40 (11.3)	28 (10.6)	22 (9.1)	
2	28 (7.9)	18 (6.8)	8 (3.3)	
3	38 (10.8)	33 (12.5)	22 (9.1)	
4	22 (6.2)	10 (3.8)	7 (2.9)	
5	7 (2.0)	3 (1.1)	0 (0.0)	
IVH, n (%)	124 (35.1)	98 (37.0)	75 (32.6)	0.591
ICH volume, median (IQR)	14.0 (5.4–38.5)	15.2 (5.0–42.4)	18.4 (5.9–42.3)	0.405
Location, n (%)			(1 missing)	0.252
Infratentorial	37 (10.5)	32 (12.1)	28 (11.6)	
Lobar	98 (27.8)	82 (30.9)	88 (36.5)	
Deep	218 (61.8)	151 (57.0)	125 (51.9)	
Taking anticoagulant, n (%)	47 (13.3)	36 (13.6)	28 (11.6)	0.761
Taking antiplatelet, n (%)	68 (19.3)	52 (19.6)	56 (23.1)	0.474
Hypertension, n (%)	157 (44.6) (1 missing)	142 (53.6)	112 (46.7) (2 missing)	0.078
Diabetes, n (%)	39 (11.1) (1 missing)	41 (15.5)	35 (14.6) (2 missing)	0.235
Systolic BP, mean (SD)	171 (34.8) (11 missing)	169 (35.4) (8 missing)	165 (33.4) (25 missing)	0.079
Onset to arrival (h), med (IQR)	2.8 (1.5–8.6)	6.7 (2.2–15.6)	5.8 (1.8–15.9)	**<0.001**
Care Process Measures				
DNR order <24 hour, n (%)	97 (27.5)	47 (17.7)	39 (16.1)	**<0.001**
Critical care admission <72 hour, n (%)	65 (18.4)	76 (28.7)	72 (29.8)	**<0.001**
Door-to-needle time for anticoagulant reversal (mins), median (IQR)	109 (67–151) (1 missing)	119 (79–199)	76 (0–163)	0.110
Door-to-target time for BP lowering (mins), median (IQR)	718 (334–1761) (17 missing)	237 (114–887) (8 missing)	138 (90–311) (3 missing)	**<0.001**
Door-to-first enteral hypertensive time (mins), median (IQR)	28 (16–47)	23 (14–43)	25 (13–44)	0.386
Repeat scan within 6 hours of END, n (%)	43/100 (43.0)	10/43 (23.3)	18/69 (26.1)	**0.020**
Palliative care <72 hours, n (%)	38 (10.8)	18 (6.8)	17 (7.0)	0.135
Outcomes				
END <48 hours, n (%)	100 (28.4) (1 missing)	43 (16.2)	69 (28.5)	**<0.001**
Died within 30 days, n (%)	99 (28.0)	57 (21.5)	38 (15.7)	**0.002**

P values ‹ 0.05 are shown in bold.

BP, blood pressure; DNR, do-not-resuscitate; END, early neurological decline; GCS, Glasgow Coma Scale; ICH, intracerebral haemorrhage; IVH, intraventricular haemorrhage.

### DNR orders

The frequency of early DNR orders (within 24 hours) was statistically different (p<0.001) across the groups (p<0.001): less common during (17.7%) and after (16.1%) implementation than before (27.5%) (table 1). Similarly, the proportion of ICH patients admitted to critical care within 72 hours was statistically different (p<0.001): increasing during (28.7%) and after (29.8%) implementation compared with before (18.4%) (table 1).

### Predictors of a DNR order

The statistically significant predictors of an early DNR order include age (OR: 1.10, 95% CI: 1.08 to 1.12, p<0.001), premorbid mRS (3 vs 0: OR: 3.48, 95% CI: 1.86 to 6.54, p<0.001; 4 vs 0: OR: 8.13, 95% CI: 3.35 to 20.71, p<0.001) and GCS on arrival (OR: 0.80, 95% CI: 0.75 to 0.86, p<0.001) (table 2). Additionally, larger ICH volume (OR: 1.01, 95% CI: 1.01 to 1.02, p<0.001) was associated with increased odds of an early DNR (table 2). The interaction between implementation of the care bundle and all baseline factors was investigated but statistically significant differences were suspected to be a by-product of existing baseline differences and not considered to be a feature of the care received.

**Table 2:** Adjusted ORs for DNR within 24 hours using multivariable logistic regression for ICH patients presenting at Salford Royal Hospital between 1 June 2013 and 31 may 2017

Predictors	Adjusted OR	95% CI	P value
(Intercept)	0.00	0.00 to 0.01	**<0.001**
Female	1.44	0.93 to 2.25	0.104
Age	1.10	1.08 to 1.12	**<0.001**
ICH Volume	1.01	1.01 to 1.02	**<0.001**
modified Rankin Scale score=1	1.44	0.72 to 2.78	0.290
modified Rankin Scale score=2	1.12	0.51 to 2.37	0.763
modified Rankin Scale score=3	3.48	1.86 to 6.54	**<0.001**
modified Rankin Scale score=4	8.13	3.35 to 20.71	**<0.001**
modified Rankin Scale score=5	1.05	0.23 to 4.51	0.948
IVH	1.40	0.80 to 2.46	0.237
Location: lobar	1.53	0.64 to 3.85	0.356
Location: deep	2.04	0.96 to 4.61	0.073
Anticoagulant	1.33	0.75 to 2.34	0.324
Systolic BP (on admission)	1.00	0.99 to 1.01	0.726
Onset to arrival (hours)	0.99	0.97 to 1.00	0.113
GCS score (on admission)	0.80	0.75 to 0.86	**<0.001**
During implementation (vs before)	0.45	0.26 to 0.75	**0.002**
After implementation (vs before)	0.49	0.28 to 0.84	**0.010**

P values ‹ 0.05 are shown in bold.

BP, blood pressure; DNR, do-not-resuscitate; GCS, Glasgow Coma Scale; ICH, intracerebral haemorrhage; IVH, intraventricular haemorrhage.

### Cardiac arrest calls

The cardiac arrest team were not called for an ICH patient before implementation but were called on five occasions overall during and after implementation. Further qualitative evaluation revealed that on only one occasion was there a cardiac or respiratory arrest with CPR performed. The events precipitating the calls included: (1) self-terminating seizure with associated vomiting and no loss of cardiac output; (2) periarrest patient, prior to ICH diagnosis; (3) cardiac arrest incorrectly diagnosed, CPR not commenced; (4) patient self-decannulated tracheostomy tube, leading to an airway emergency and prolonged cardiorespiratory arrest, successfully resuscitated; (5) cardiac arrest team called in error for a patient with a DNR order; CPR not commenced.

### Care associated with DNR orders

Early DNR orders were associated with a reduced odds of escalation to critical care (OR: 0.07, 95% CI: 0.03 to 0.17, p<0.001). Commencement of palliative care within 72 hours was far more likely (OR: 8.76, 95% CI: 4.74 to 16.61, p<0.001) if an early DNR was in place. There was no evidence to suggest an association between early DNR and door-to-needle time for anticoagulant reversal, door-to-target time for BP lowering, door-to-first enteral hypertensive time, nor rescanning within 6 hours of END (table 3).

**Table 3:** Adjusted associations between DNR within 24 hours and ICH care processes

Care process outcome	Model type	Estimate for DNR <24 hours	95% CI	P value	Additional model variables
Door-to-needle time anticoagulant reversal (hour)	Linear regression	0.49	−0.58 to 1.56	0.365	
Door-to-target time BP lowering (hour)	Log-linear regression	−0.22	−0.52 to 0.08	0.146	Age, ICH Volume, IVH, ICH Location, GCS, Systolic BP on admission
Door-to-first enteral hypertensive time (hour)	Log-linear regression	0.18	−0.07 to 0.43	0.147	Age, ICH Volume, IVH, ICH Location, GCS
Admission to critical care in first 72 hours	Logistic regression	0.07	0.03 to 0.17	**<0.001**	Age, ICH Volume, IVH, ICH Location, GCS, pre-ICH mRS
Repeat scan within 6 hours of END	Logistic regression	0.61	0.27 to 1.35	0.222	Age, ICH Volume, IVH, ICH Location, GCS, pre-ICH mRS
Commencement of palliative care <72 hours	Logistic regression	8.76	4.74 to 16.61	**<0.001**	Age, ICH Volume, IVH, ICH Location, GCS, pre-ICH mRS

All models adjusted for onset to arrival and implementation group (before, during or after). Additional variables that were adjusted for are detailed. All variables were chosen clinically.

P values ‹ 0.05 are shown in bold.

BP, blood pressure; DNR, do-not-resuscitate; END, early neurological decline; GCS, Glasgow Coma Scale; ICH, intracerebral haemorrhage; IVH, intraventricular haemorrhage; mRS, modified Rankin Scale.

## Discussion

The main findings of this study are (1) early DNR orders were more likely with older age, lower GCS on arrival, premorbid mRS of 3–4, deep ICH location and larger ICH volume; (2) early DNR orders were less common after ABC bundle implementation (16.1% vs 27.5%), but we found no evidence that this led to harm through futile, undignified and potentially traumatic resuscitation attempts.

Our finding that DNR orders are independently associated with less aggressive supportive care is compatible with previous studies. Early DNR orders are common after ICH,^13^ are independently associated with poor outcomes, and have previously been associated with a reduction in the overall level of aggressiveness of ICH care,^13^ a lower number of stroke unit admissions and less deep vein thrombosis prophylaxis.^14^ A multicentre prospective observational study has tested a policy of avoiding DNR orders in first 5 days of care in a subgroup of patients with severe ICH (defined as GCS score ≤12) and shown a considerable improvement in observed case fatality (vs ICH score predicted case fatality), with an mRS of 0–3 at 90 days in nearly a third.^15^ Our current analysis of ABC bundle implementation suggests that the observed reduction in early DNR orders is part of a wider increase in the level of supportive care.

A particular strength of this study is a large sample of consecutive ICH patients, representative of routine clinical care. The ABC-ICH study provides a natural experiment in which a quality improvement intervention targeting ICH-specific care indirectly led to reduced DNR orders, which in turn partially mediated a reduction in case fatality. Furthermore, detailed information on premorbid health and disease severity, including imaging characteristics, allowed for adequate adjustment in regression models and comprehensive and complete data collection was possible as all records have been on a single EPR since 2013.

The reduction in DNR orders was associated with a reduction in 30-day mortality. This relationship is, however, likely to be multifactorial. Specifically, our data showed a reduction in likelihood of escalation to critical care and greater likelihood of commencement of palliative care within 24 hours, aligning with prior data in ICH patients.^13^ These mechanistic findings can be translated to three potential improvements which could improve quality of care:

1. The decision to instigate a DNR order should be based on multiple factors which should include (based on our study data) but not be limited to: age, GCS on arrival, pre-morbid mRS, location of ICH and size of ICH.
2. In this instance that adverse findings associated with these factors are present, that is, older age, lower GCS on arrival, premorbid mRS of 3–4, deep ICH location and larger ICH volume—the inability or delay in instigating a DNR order should not be met with an expectation of harm occurring.
3. Clinicians should consider factors described above, communicate with patients and relatives and provide aggressive supportive care early in order to maximise the possibility of a better outcome if immediate deterioration is not expected/predicted.

Implementation of the ABC care bundle was associated with improved survival, partially mediated by less early DNR orders. Reassuringly, we have shown that this was not associated with an increase in resuscitation attempts, but DNR orders were independently associated with less aggressive supportive care. In line with current American Heart Association/American Stroke Association guidance,^9^ clinicians should use DNR orders judiciously in acute ICH patients and ensure they do not lead to limitations of care beyond their intended purpose.

## Limitations

First, this is a retrospective study which considers patients from a single centre, meaning we are not able to make causal inferences. Second, ICH patients with an mRS of four and above may be under-represented in the analysis, given the small numbers observed in the ‘during’ and ‘after’ periods. Further, there were significant differences between the ‘before’, ‘during’ and ‘after’ populations which could indicate confounding. Although we adjusted for baseline variables, a larger study would be needed to validate our findings. Third, there are often quality improvement projects implemented within the centre, which may have caused changes to procedures during the study which we were unable measure. Finally, we were limited to 30-day mortality and are not able to understand the association with a change in disability from the reduction in early DNRs.
